# A β-Catenin-Dependent Wnt Pathway Mediates Anteroposterior Axon Guidance in *C. elegans* Motor Neurons

**DOI:** 10.1371/journal.pone.0004690

**Published:** 2009-03-04

**Authors:** Géraldine S. Maro, Matthew P. Klassen, Kang Shen

**Affiliations:** 1 Howard Hughes Medical Institute, Department of Biology and Pathology, Stanford University, Stanford, California, United States of America; 2 Neurosciences Program, Stanford University, Stanford, California, United States of America; Institut de la Vision, France

## Abstract

**Background:**

Wnts are secreted glycoproteins that regulate diverse aspects of development, including cell proliferation, cell fate specification and differentiation. More recently, Wnts have been shown to direct axon guidance in vertebrates, flies and worms. However, little is known about the intracellular signaling pathways downstream of Wnts in axon guidance.

**Methodology/Principal Findings:**

Here we show that the posterior *C. elegans* Wnt protein LIN-44 repels the axons of the adjacent D-type motor neurons by activating its receptor LIN-17/Frizzled on the neurons. Moreover, mutations in *mig-5*/Disheveled, *gsk-3*, *pry-1*/Axin, *bar-1*/β-catenin and *pop-1*/TCF, also cause disrupted D-type axon pathfinding. Reduced BAR-1/β-catenin activity in D-type axons leads to undergrowth of axons, while stabilization of BAR-1/β-catenin in a *lin-23*/SCF^β-TrCP^ mutant results in an overextension phenotype.

**Conclusions/Significance:**

Together, our data provide evidence that Wnt-mediated axon guidance can be transduced through a β-catenin-dependent pathway.

## Introduction

During nervous system development, neurons and growth cones migrate extensively to build neural circuits. Conserved guidance molecules important for these migrations have been identified in a variety of model organisms [1]. Several morphogens, or secreted molecules that pattern the embryo, have also been demonstrated to serve as axon guidance cues later in development [2]. The Wnt family of secreted glycoproteins, in addition to its roles in axis and fate specification, also regulate axon guidance. In the *Drosophila* central nervous system DWnt5 repels axons expressing the receptor tyrosine kinase Derailed from posterior commissures [3]. Likewise, mouse corticospinal axons utilize Ryk, the Derailed orthologue, to navigate away from Wnts [4], [5]. Wnts can also act as axonal attractants, as was demonstrated for commissural axons whose attraction to Wnt4 is mediated by the Frizzled receptor Fz3 [6].

Whereas substantial research has implicated a variety of Wnts and their receptors in the regulation of axon guidance, our understanding of the downstream signaling pathways remains limited [7], [8]. Three different intracellular pathways are known to function downstream of Wnts (reviewed in [9]): the β-catenin-dependent canonical pathway, the planar cell polarity (PCP) pathway and the Wnt/calcium pathway. In the canonical pathway, β-catenin is phosporylated and targeted for proteasome degradation by the glycogen synthase kinase-3 (GSK-3)/Axin/Adenomatous polyposis coli (APC) complex. Upon binding of Wnt to its Fz receptor, the cytosolic protein Disheveled (Dsh) inhibits GSK-3 and thus allows accumulation of β-catenin and its translocation into the nucleus, where it activates transcription of target genes together with transcription factors of the the T-Cell Factor (TCF) family.

Despite extensive characterization of each signal transduction pathway in many Wnt-dependent processes, the contribution of these pathways during axon guidance is not well understood. GSK-3 was shown to regulate axon cytoskeletal remodeling via phosphorylation of microtubule-associated protein MAP1B, defining a proposed divergent canonical pathway [10]. There is also evidence for the PCP pathway mediating axon pathfinding. Mice mutant for *Fz3^−/−^* and *Celsr3^−/−^*, a mammalian orthologue of *Drosophila flamingo*, exhibit similar defects in several axonal tracts (including the internal capsule and the anterior commissure) [11]. In addition, the small GTPases Rho and Rac/Cdc42 can affect the stability of the actin and microtubule cytoskeleton [12], and Wnt7b, Dsh, Rac and c-Jun N-terminal Kinase (JNK) have been shown to increase dendritic branching in cultured hippocampal neurons [13]. The Wnt/calcium pathway, which is activated in motile and metastatic cells [14], is also a good candidate for regulating axon guidance, a process that can be thought of as specialized directed movement. Indeed, a recent study demonstrated a role for atypical PKC signaling in mediating commissural axons attraction to Wnt in the spinal cord [15].

In *C. elegans*, Wnts regulate the anteroposterior guidance of A/PVM axons [16], [17], suggesting that Wnts are evolutionarily conserved axon guidance molecules. Given the abundance of genetic reagents available in the Wnt pathways, *C. elegans* is an excellent model system to examine the contributions of the alternative downstream components to Wnt/Fz-mediated axon guidance. In this study we show that the posterior D-type motor axons utilize Wnts as repulsive cues to terminate at the appropriate position along the anteroposterior (AP) axis. This is mediated cell-autonomously by a Fz receptor, and is partially transduced through a β-catenin-dependent pathway.

## Materials and Methods

### Strains & Genetics

Wild-type animals were of Bristol variety N2 strain. Strains were maintained using standard methods at 20°C. Some strains were provided by the Caenorhabditis Genetics Center (CGC).

### Transgenic lines

*wyIs75* [*Punc-47::dsred*; *Pexp-1::gfp*; *Podr-1::dsred*], *wyEx792* [*Plin-44::signal sequence::flag::gfp::lin-44*; *Podr-1::gfp*] and *wyEx1107* [*Pegl-20::lin-44*; *Podr-1::gfp*] [18], *wyEx1186* [*Punc-47::lin-17*; *Podr-1::gfp*], *wyEx1444* [*Punc-129Δ::lin-44*; *Podr-1::gfp*] [18], *wyEx1607* [*Punc-47::lin-17::yfp*; *Pttx-3::mCherry*], *wyEx1634* and *wyEx1696* [*Punc-47::bar-1::gfp*; *Podr-1::gfp*], *wyEx2062* [*Punc-25::lin-23*; *Podr-1::gfp*].

### Mutants

*LGI*, *lin-44(n1792)*, *lin-17(n671)*, *mom-5(ne12) dpy-5(e61)*, *mig-1(e1787)*, *gsk-3(nr2047)*, *pry-1(mu38)*, *pop-1(q645)*; *LGII*, *cwn-1(ok546)*, *dsh-1(ok1445)*, *dsh-2(or302)*, *cam-1(gm122)*, *mig-5(rh94)*, *mig-5(rh147)*, *lin-23(ot1)*, *lin-23(e1883)*; *LGIV*, *egl-20(n585)*, *cwn-2(ok895)*; *LGV*, *cfz-2(ok1201)*, *mom-2(or77)*; *LGX*, *lin-18(e620)*, *bar-1(ga80)*.

### Molecular Biology

*Pexp-1::gfp* was made by amplifying a 3.5 kb EcoRV–EcoRV fragment from the *exp-1* promoter with primers containing an upstream PstI site and a downstream BamHI site. This fragment was then cloned into pPD95.75 (A. Fire) using PstI and BamHI. *Punc-47::dsred* was made by amplifying a 1.4 kb promoter region upstream of the start codon with primers containing an upstream HindIII site and a downstream KpnI site. This fragment was then cloned into a dsRed-containing plasmid. All other expression clones were made in the pSM vector, a derivative of pPD49.26 (A. Fire) with extra cloning sites (S. McCarroll and C.I. Bargmann, personnal communication). To generate *Punc-47::lin-17* and *Punc-47::lin-17::yfp* respectively, the *lin-17* and *lin-17::yfp* inserts [18] were ligated into a *Punc-47* pSM vector using AscI/KpnI. *Punc-47::bar-1::gfp* was made by subcloning a full-length *bar-1* cDNA, fused to GFP at the C-terminus, insert [19] into a *Punc-47* pSM vector using AscI/ApaI. The *lin-23* cDNA was PCR amplified from the *yk784a08* cDNA clone. One point mutation was found and corrected. The insert was then ligated into a pSM vector containing 1.8 kb of the *unc-25* proximal promoter, using KpnI. Detailed cloning and promoter information is available upon request.

### Transgenic Lines and Fluorescence microscopy

Constructs were injected into the gonad of adult animals at concentrations of 15 ng/µl (*Punc-47::dsRed*, *Punc-47::lin-17*, *Punc-47::bar-1::gfp*), 50 ng/µl (*Pexp-1::GFP*, *Punc-129Δ::lin-44*), 1 (*Plin-44::signal sequence::flag::gfp::lin-44*, PCR product), 0.5 ng/µl (*Punc-47::lin-17::yfp*), 5 ng/µl (*Punc-25::lin-23*), respectively, with 20–40 ng/µl of either *Podr-1*::*dsred*, *Podr-1*::*gfp* or *Pttx-3::mCherry* as the co-injection marker. Multiple transgenic lines for each transgene were examined for fluorescence expression, rescue and/or localization patterns. Fluorescence images were obtained using Zeiss LSM 510 META. Except when stated otherwise in the text, all animals scored were at the L4 stage.

### Statistics

A Fisher exact test was used to evaluate statistically significant differences between various genetic backgrounds.

## Results

### Wnts are required for correct axon guidance of D-type motor neurons

**Figure 1:**
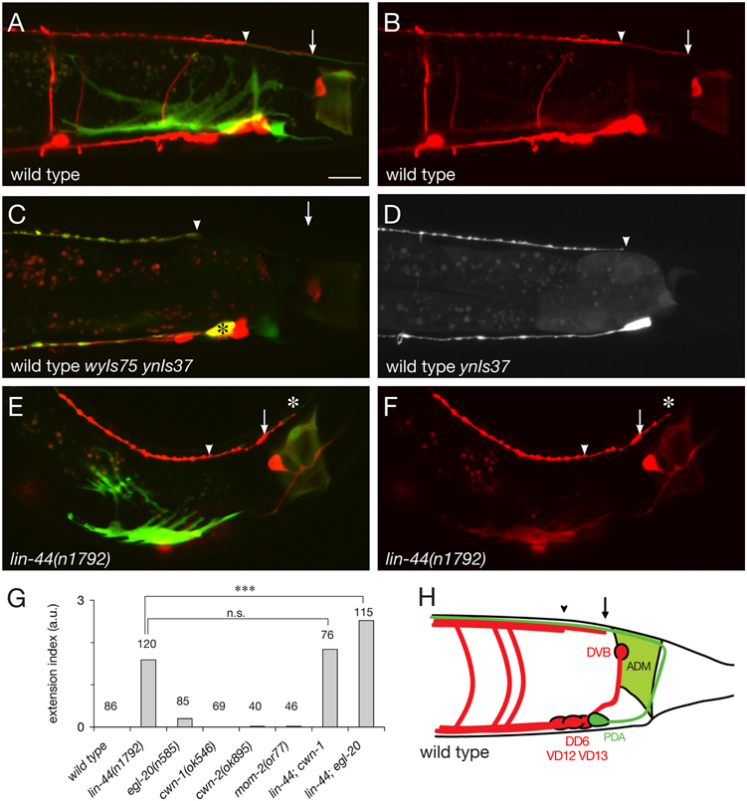
Posterior D-type axons are misguided in *lin-44* mutants. (A,B) The posterior-most D-type axons, DD6, VD12 and VD13, visualized with DsRed in *wyIs75* animals, terminate at stereotyped positions along the dorsal nerve cord. A bundle of axons terminates at the anteroposterior position corresponding to where these cell bodies are located (arrowhead), and is extended by a process (arrow) that reaches the anterior border of the anal depressor muscle (ADM) labeled with GFP in (A). (C,D) DD6 axon is labeled with a *Pflp-13::GFP* (*ynIs37*) and terminates anterior to VD13 axon. (E,F) D-type axons overextend (asterisk) past the ADM into the tail in *lin-44* mutant animals. The arrow and arrowhead point termination positions of the thick bundle and thin process in wild-type animals, respectively. (G) Overextension index for mutants in the three Wnts expressed in the tail region, compared to those of wild-type animals. *lin-44*, but not *egl-20*, *cwn-1*, *cwn-2 and mom-2* single mutants, show a dramatic axon guidance defect. The severity of the phenotype is enhanced in the *lin-44*; *egl-20* double mutant. Scale bar, 10 µm. (H) Schematic diagram of D-type axons (red) and ADM (green) in the tail region, as seen in *wyIs75* animals. Other cells labeled in *wyIs75* animals include the intestinal muscles (on each side of the intestine, not depicted), PDA and DVB neurons. a.u.: arbitrary units.

The GABAergic D-type motor neurons – 6 DD and 13 VD neurons - innervate body wall muscles and are responsible for reciprocal inhibition during locomotion of *C. elegans* [20]. Their cell bodies are found along the ventral midline. Each of these neurons extends an axon anteriorly; this process then bifurcates dorsally to form a commissure, and bifurcates again in the dorsal nerve cord where it grows both posteriorly and anteriorly [21] (Fig. 1). We visualized these axons in *wyIs75* animals using a *Punc-47::dsRed* reporter that labels all GABAergic neurons (Fig. 1A,B). In the dorsal nerve cord, the DD processes fasciculate with those of the VD neurons and terminate at stereotyped positions along the anteroposterior axis. The termination points for the posterior neurons were determined from confocal micrographs of *Punc-47::dsRed* transgenic animals (Fig. 1). The most posterior DD and VD processes extend as a bundle that terminates ∼20 µm anterior to the anal depressor muscle (ADM, labeled with GFP in *wyIs75* animals), at the anteroposterior position corresponding to the locations of the DD6, VD12 and VD13 cell bodies, which reside on the ventral side (Fig. 1A,B, arrowhead). In the vast majority of animals (79%), this thick bundle becomes thinner and extends further posteriorly to reach the anterior border of the ADM (Fig. 1A,B,H, arrow). In the dorsal nerve cord, neurons of a given class tile their processes resulting in the fasciculation of only one DD and one VD process at any given AP position [21]. Therefore the thick bundle consists of DD6 and VD13 axons, and the thin bundle is formed by one of these two only. To further determine the identity of each axon, we examined a DD-specific fluorescent line, carrying a *Pflp-13::GFP* transgene (*ynIs37*). We found that the DD6 posterior process in the dorsal nerve cord terminates at the anteroposterior location corresponding to its ventrally localized cell body (Fig. 1C,D), and thus that the thick bundle corresponds to DD6 and VD13 axons, prolonged posteriorly by VD13 axon only.

Three members of the Wnt family of secreted glycoproteins – LIN-44, EGL-20 and CWN-1 – are expressed in the *C. elegans* tail [16] and have been shown to regulate asymmetric cell divisions [22], [23], cell migration [16], [24], [25], neuronal polarity [26], axon guidance [16], [17] and synapse patterning [18]. We asked whether these Wnts could also play a role in defining the posterior termination point of D-type axons in the dorsal nerve cord. We found that in 83% of *lin-44*/Wnt mutants the D-type axons extended further posteriorly in the tail as compared to wild-type animals (Fig. 1E,F). In order to quantify the overextension phenotype, we calculated an extension index that binned three possible phenotypes of *lin-44* animals: 1) in mildly affected animals, the relative positions of DD6 and VD13 are correct, but they both terminate up to 5 µm further posteriorly than in wild-type animals (light blue in wFig. S1); 2) in intermediate cases, DD6 axon reaches the anterior border of the ADM (median blue in Fig. S1); 3) in the most severe cases, DD6 and/or VD13 overextends past the anterior border of the ADM (dark blue in Fig. S1; Fig. 1E,F). The extension index was calculated by assigning a weight score of +1, +2, or +3, to the mild, intermediate and severe category of phenotypes, respectively (Fig. S1). A wild-type phenotype was given a score of 0. Thus, if all the animals of a particular genotype displayed the most severe phenotype (+3), the index would be +3. Similarly, if half of the population displayed a mild phenotype (+1) and the other half displayed an intermediate phenotype (+2), the index would be +1.5. The frequency of these different phenotypes in *lin-44* mutants is presented in Figure S2. In contrast to *lin-44* animals, which have a +1.6 overextension index, *egl-20* mutants show only a subtle overextension defect (+0.2) (Fig. 1G). However, *lin-44*; *egl-20* double mutants have a more dramatic phenotype than *lin-44* single mutants, with an overextension index of +2.5. *cwn-1* mutants have normal axon guidance of the D-type neurons, as do the remaining two Wnt mutants, *cwn-2* and *mom-2*, and the *cwn-1* mutation does not increase the severity of the *lin-44* mutant phenotype (Fig. 1G, Fig. S2). Together these data suggest that the two Wnts LIN-44 and EGL-20 act cooperatively to regulate D-type axon guidance.

### The Frizzled receptor LIN-17 regulates axon guidance cell-autonomously in the D-type neurons

**Figure 2:**
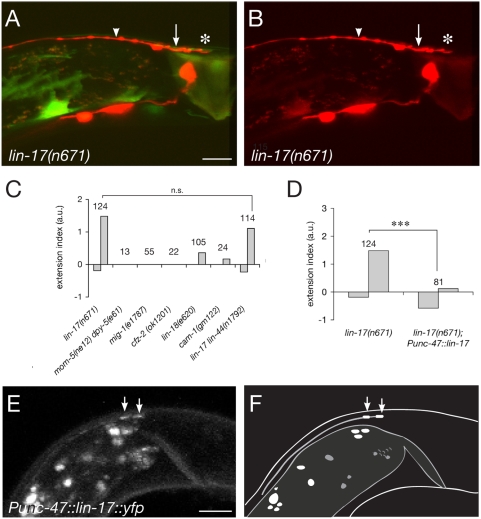
LIN-17 Fz receptor mediates the response to Wnts in D-type axons. (A,B) *lin-17* mutant animals show a phenotype similar to that of *lin-44* mutants, with axons terminating posterior (asterisk) to their normal positions (arrow and arrowhead). (C) Extension index for mutants in each known Fz orthologue (*lin-17*, *mom-5*, *mig-1*, *cfz-2*), in the Ryk/Derailed orthologue *lin-18*, and in the tyrosine kinase Ror2 homologue *cam-1*. Mild overextension phenotypes were observed in less than 30% and 15% of the *lin-18* and *cam-1* mutant animals, respectively. No increase in the severity of the extension phenotype of *lin-17* mutants was observed in *lin-17*; *lin-44* double mutants. (D) Comparison of the extension index of *lin-17* mutants and *lin-17* mutants carrying a *Punc-47::lin-17* transgene. p<0.001. (E) Fluorescent distribution pattern of a LIN-17::YFP fusion protein is enriched at the posterior tip of D-type axons, here shown for an L1 animal. (F) Schematic diagram corresponding to the confocal image shown in (D). Scale bars, 10 µm (A,B) and 5 µm (E). a.u.: arbitrary units.

Both seven-transmembrane Frizzled receptors and the Ryk/Derailed tyrosine kinase receptor have been implicated in Wnt-mediated axon guidance [3]–[6], [27], [28]. The *C. elegans* genome encodes four Frizzled receptors, LIN-17, MIG-1, MOM-5 and CFZ-2, and one Ryk/Derailed homologue, LIN-18. We therefore tested their requirement by analyzing axon guidance phenotypes for the corresponding mutants. We found that D-type neurons showed severe axon guidance defects in *lin-17* mutant animals (Fig. 2A–C). Interestingly, loss of *lin-17* function results in both overextension (61% of the animals; +1.5) and underextension (34% of the animals; −0.2) phenotypes (Fig. 2C and Fig. S3). The over- and underextension indices were calculated independently from over- and underextended animals, respectively (see Fig. S1 and Fig. S3 for more detail). In contrast, the three other Frizzled mutants have no significant axon guidance defects of the D-type neurons (Fig. 2C). It should be noted however that as *mom-5* mutations cause a maternal effect embryonic lethal phenotype, the lack of D-type guidance defects in animals from heterozygous mothers could be because of maternal rescue. Finally, *lin-18* mutants, as well as the Wnt receptor tyrosine kinase Ror2 homologue *cam-1* mutants [29], show only a subtle overextension phenotype (Fig. 2C), and the *lin-18* mutation did not enhance significantly the overextension phenotype of *lin-17* mutants (Fig. S3).

Because Wnt signaling is known to affect a variety of cells in the *C. elegans* tail, it is possible that changes in the environment of the D-type neurons affect guidance of their axons. Alternatively, *lin-17* function could be required cell-autonomously in the D-type neurons. To distinguish between these possibilities, we expressed functional LIN-17 in the D-type neurons and in 7 other GABAergic neurons (the RME's, AVL and RIS in the head, and DVB in the tail, none of which send processes into the dorsal nerve cord) in otherwise *lin-17* mutant animals. We found that this transgene completely rescued the axon guidance defect (Fig. 2D), suggesting that Wnt signaling is required cell-autonomously in the D-type neurons to regulate axon guidance. In addition, to verify that LIN-17 acts downstream of Wnt/LIN-44 in the same genetic pathway, we constructed the double mutant *lin-44(n1792) lin-17(n671)* which showed a phenotype indistinguishable from *lin-17* single mutant animals (Fig. 2C).

If Wnt is acting as an axon guidance molecule, one might expect its receptor to localize at axonal growth cone. We therefore examined the LIN-17 subcellular localization in the DD-VDs by expressing a functional, fluorescently tagged LIN-17::YFP. Unfortunately, signal from this fusion protein was too faint to allow examination of D-type axons at the time of outgrowth in embryos (DD's) or L1/L2 animals (VD's). Nevertheless, we found that LIN-17::YFP was enriched at the posterior tip of the most posterior D-type axon (the only one that can be unambiguously identified within the dorsal nerve cord) (Fig. 2E,F). This localization appears to be unchanged in *lin-44*; *egl-20* animals, and is thus Wnt-independent (data not shown). Together these data raise the possibility that local activation of LIN-17/Frizzled at axon growth cones by the Wnts LIN-44 and EGL-20 might ensure accurate D-type axon guidance along the AP axis in *C. elegans*.

### Wnts are instructive guidance cues for the D-type axons

One potential model for the action of Wnts on D-type axons is that Wnts directly act as guidance cues, as suggested by studies on commissural axons in mice [6]. Alternatively, Wnts could be permissive factors that enable these axons to respond to an undetermined axon guidance molecule, possibly by regulating D-type neuronal fate. To distinguish between these possibilities, we used a partial *unc-129* promoter [30] to drive the expression of LIN-44/Wnt in dorsal body wall muscles in *lin-44*; *egl-20* mutant animals. If the instructive hypothesis is correct, we expect that Wnt would repel anterior D-type axons locally because LIN-44 is now expressed by the adjacent body wall muscles. Alternatively, if Wnt is acting as a permissive factor, this manipulation would not affect the directionality of the instructive cue, resulting in normal guidance of the anteriorly-directed D-type processes. To control for possible effects caused by overexpression of Wnt, we also used the *lin-44* endogenous promoter to express LIN-44 in epithelial cells at the tip of the tail.

**Figure 3:**
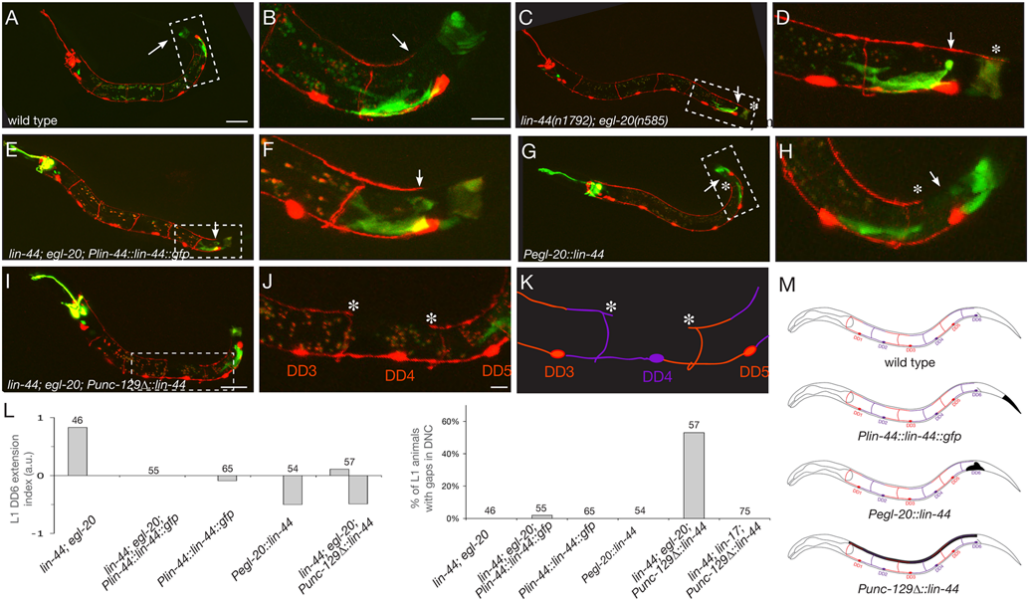
Wnts act instructively to repel posterior axons. (A,B) In wild-type animals, the dorsal posterior branch of DD6 extends up to the position corresponding to that of its cell body in the VNC (arrow). (B) is a higher magnification view of the boxed region in (A). (C,D) In *lin-44*; *egl-20* mutants, the dorsal posterior process of DD6 overextends (asterisk) as compared to its normal position (arrow). (D) is a higher magnification view of the boxed region in (C). (E,F) Expression of Wnt in the posterior tail using a *Plin-44::lin-44::gfp* array rescues the DD6 overextension phenotype in *lin-44*; *egl-20* mutant animals. (F) is a higher magnification view of the boxed region in (E). (G,H) Expression of Wnt in a domain slightly anterior to the *lin-44* expression domain using a *Pegl-20::lin-44::gfp* array rescues the DD6 overextension phenotype in *lin-44*; *egl-20* mutant animals, and causes underextension defects in 50% of the animals. (H) is a higher magnification view of the boxed region in (G). (I–K) Ectopic expression of Wnt in the dorsal body wall muscles causes DD processes to stop prematurely (asterisks), resulting in gaps in the DNC. (J) is a higher magnification view of the boxed region in (I). (K) is a schematic corresponding to the confocal image shown in (J). (L) Quantification of the DD6 extension phenotypes (top) and the DNC gap phenotypes (bottom) observed in L1 animals expressing no Wnt, Wnt in the posterior tail, or Wnt in the dorsal body wall muscles in mutant or wild-type backgrounds. (M) Schematic showing DD morphology in L1 animals and the domain of Wnt expression expected from the rescuing and ectopic expression arrays. Scale bars, 20 µm (A,C,E,G,I), 10 µm (B,D,F,H) and 5 µm (J).

In order to reliably score individual axons, we examined L1 animals because only the 6 DD, but not the VD neurons, have developed at this stage. In wild-type animals, the DD6 dorsal process terminates at the AP position of its cell body (Fig. 3A,B). In *lin-44*; *egl -20* animals 80% of DD6 processes overextend into the tail past the ADM (Fig. 3C,D,L). As expected, expressing a *Plin-44::lin-44::gfp* construct was able to completely rescue this defect (Fig. 3E,F, L,M). Using the *egl-20* promoter, which is expressed in a domain slightly more anterior than *lin-44*, led to an underextension of DD6 posterior process in 50% of the animals (Fig. 3G,H,L). However, when LIN-44 was expressed in the dorsal body wall muscles, using a *Punc-129Δ::lin-44* construct (Fig. 3M), not only did we observe that DD6 axon guidance was robustly rescued or underextended, but also that DD4 or DD5 displayed an underextension phenotype in 54% of the animals, resulting in a gap in the dorsal nerve cord (Fig. 3I–L). This was never observed in animals expressing Wnt posteriorly from the endogenous *lin-44* promoter, nor from the *egl-20* promoter, both of which direct expression in a domain posterior to the DD-VD's axons (Fig. 3L,M). When we examined these dorsal axon gaps closely, we found that the gaps were often caused by the undergrowth of two adjacent axons. An example of such a gap is shown in Figure 3I–K. In this animal, both the posterior process of DD4 and the anterior process of DD5 are underextended, giving rise to a gap (Fig. 3I–K). If Wnts primed the DD neurons to respond to another axon repellent, we would expect that repellent to form a posterior to anterior gradient because loss of Wnts causes the axons to overextend posteriorly. The underextension of the DD5 anterior process is not consistent with Wnt playing a permissive role by regulating the response of D-type axons to an independent AP guidance cue. Instead, our data support an instructive model in which Wnts secreted from the tail act locally as repulsive cues to promote D-type axon termination.

This model predicts that increasing the levels of Wnt produced in the tail should cause DD6 axon to stop prematurely. We tested this prediction by re-introducing wild-type *lin-44* and *egl-20* alleles in animals carrying the *Plin-44::lin-44::gfp* rescuing construct, and comparing the extent of DD6 outgrowth with the mutant background. Consistent with our model, we found that animals expressing Wnt both from endogenous loci and from the transgene show a mild but significant underextension phenotype (10% of the animals), whereas transgenic mutant animals do not (Fig. 3L).

Finally, since our analysis of Frizzled mutants suggests that the LIN-17 receptor is responsible for mediating this Wnt-induced repulsion, we asked if the repulsion observed in our Wnt-overexpressing line was dependent on the presence of LIN-17. Consistently with this prediction, we found that a null mutation in the *lin-17* gene completely suppressed the outgrowth defects seen in animals carrying the *Punc-129Δ::lin-44* transgene (Fig. 3L).

### Mutants in the canonical Wnt pathway also show defects in D-type axon guidance

Wnts have been shown to act as axon guidance molecules in *Drosophila* [3] and vertebrates [4]–[6], [27], but the downstream effectors involved in this process are only starting to be identified [15]. *C. elegans* provides an attractive model to dissect these downstream signaling pathways since mutants have been isolated in both the canonical and non-canonical Wnt pathways. We began our analysis by examining mutants for the canonical pathway, which is well conserved among worms and other organisms [31].

**Figure 4:**
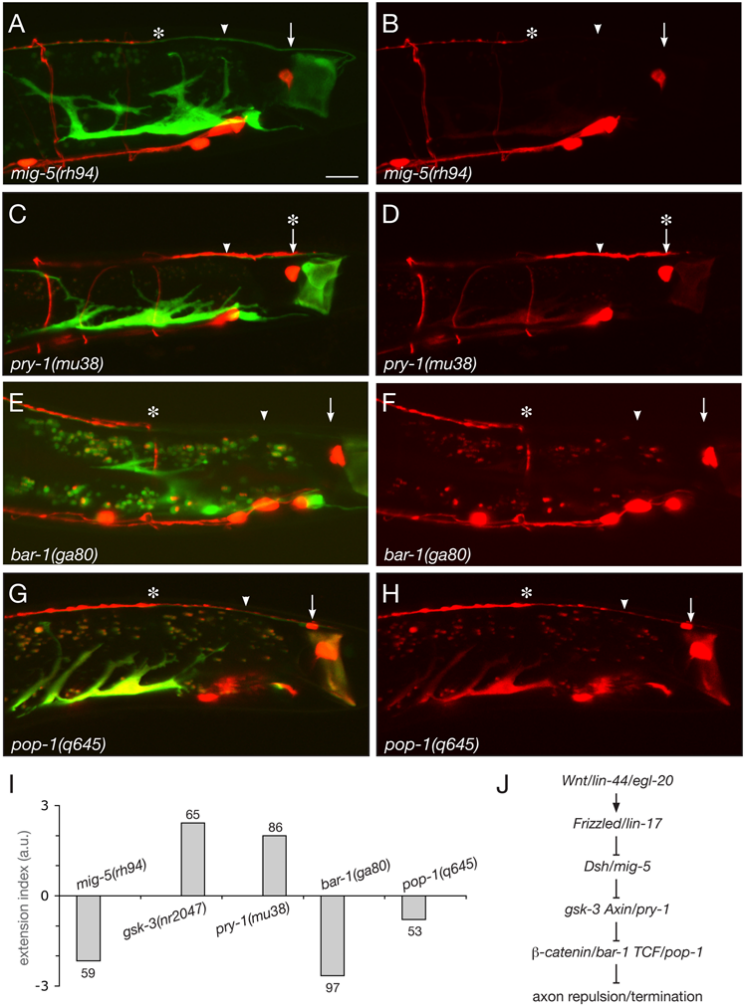
Mutants in the canonical Wnt pathway display D-type axon guidance defects. (A–H) Posterior D-type axons underextend in *mig-5*/Dsh mutants (A,B), *bar-1*/β-catenin mutants (E,F) and *pop-1*/TCF mutants (G,H), but overextend in *pry-1*/Axin mutants (C,D). In all images, the arrow and arrowhead indicate the wild-type termination points, and the asterisk indicates the abnormal termination point of posterior D-type axons. (I) Extension index for mutants in components of the canonical Wnt pathway. (J) Genetic pathway controlling D-type axon guidance, inferred from mutant analysis. Note that Dsh acts negatively in this pathway, opposite to its predicted role in the canonical pathway. Scale bar, 10 µm. a.u.: arbitrary units.

In the canonical pathway, Wnt binding to Fz activates the cytosolic protein Dsh, which inactivates the destruction complex formed by GSK-3, Axin, and the adenomatous polyposis coli (APC) protein. This leads to stabilization of β-catenin and its translocation to the nucleus where it can activate target genes together with TCF-LEF transcription factors (reviewied by [32]. The *C. elegans* genome encodes three Dsh orthologues: DSH-1, DSH-2, and MIG-5. We found that 95% of *mig-5*/Dsh mutants displayed guidance defects of the D-type axons (Fig. 4A,B and I). However, opposite to the predicted positive action of Dsh in the canonical Wnt pathway, *mig-5*/Dsh mutant animals showed a strong underextension phenotype (−2.2; Fig. 4A,B,I,J and Fig. S4), suggesting that Wnt/Fz signaling inhibits Dsh function in this pathway. *dsh-1* mutants also showed an underextension phenotype, although much weaker and less penetrant than *mig-5*/Dsh mutants. *dsh-2* mutants did not show any defect (Fig. S4). The molecular mechanism underlying the antagonistic action of LIN-44/Wnt and MIG-5/Dsh is currently unclear.

If Wnts were acting through a β-catenin-dependent pathway, we would expect components of the destruction complex to be required for axon termination, and β-catenin and TCF to be instead required for axon elongation. Indeed, both *gsk-3* and *pry-1*/Axin mutants show defects similar to *lin-44*/Wnt mutants, with 97% and 98% of the animals having overextended D-type axons (+2.4, +2.7), respectively (Fig. 4C,D,I and Fig. S4). The *C. elegans* genome encodes four β-catenin homologues [33] – BAR-1, WRM-1, HMP-2 and SYS-1 [34]. In contrast to the single vertebrate β-catenin that assumes multiple functions in transcription and cell adhesion, the four worm β-catenins appear to have more specialized roles [35]. Because *bar-1* has been implicated in the canonical Wnt pathway that regulates Q cell migration [25], [35], we first analyzed D-type axon guidance in *bar-1* mutants. As expected, *bar-1*, as well as *pop-1*/TCF mutants show underextension defects (100% and 53% of the animals, −2.7 and −0.8, respectively; Fig. 4E–I and Fig. S4). In all of the above-mentioned mutants, the commissures of the posterior D-type neurons were found to reach the dorsal nerve cord normally, and the position of their cell bodies was similar to wild type in the vast majority of the animals, arguing against the possibility that the observed axon guidance defects were secondary to other morphological defects. Finally, we examined DD6 axon guidance in the above-mentioned mutants at the L1 stage and found that all showed extension defects consistent with the L4 stage phenotypes (Fig. S5). Together our results indicate that a β-catenin-dependent pathway regulates guidance of D-type axons along the AP axis (Fig. 4J).

### BAR-1/β-catenin is required cell-autonomously for axon guidance and does not act by controlling the fate of D-type neurons

Disheveled, Axin and the GSK-3 kinase have previously been shown to regulate axon remodeling through a β-catenin-independent pathway [10], [36]. Our findings suggest that a β-catenin-dependent pathway could also play a role in axon guidance. We therefore sought to examine the requirement for β-catenin during D-type axon pathfinding in more detail.

**Figure 5:**
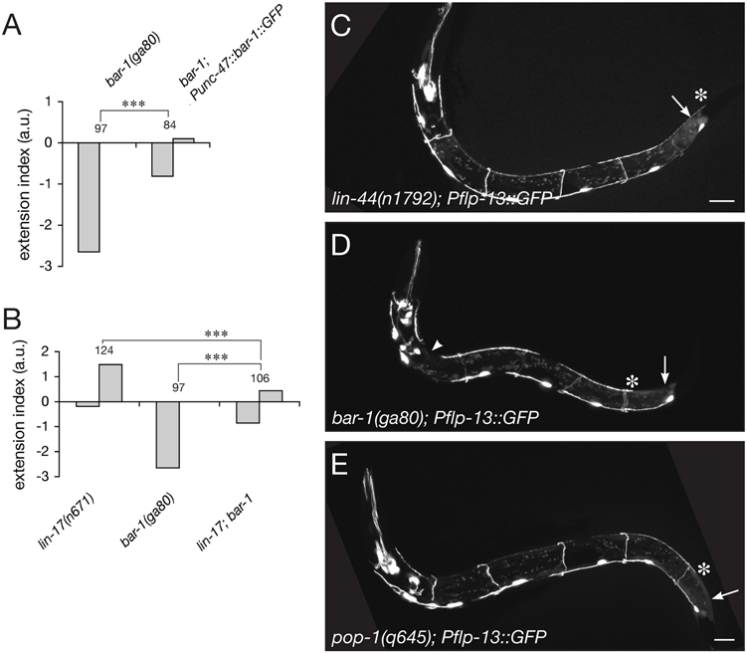
BAR-1/β-catenin regulates axon pathfinding cell-autonomously in D-type neurons. (A) Expression of a functional BAR-1::GFP fusion protein in D-type neurons is sufficient to partially rescue the underextension defect observed in *bar-1(ga80)* animals. (B) Extension index of *lin-17*; *bar-1* double mutants, compared to either of the single mutants. The double mutant shows a phenotype intermediate between the two single mutants. ***p<0.001. (C–E) Expression of a *Pflp-13::GFP* reporter (*ynIs37*) is not affected in *lin-44* (C), *bar-1* (D) and *pop-1* (E) mutants DD neurons. Scale bar, 10 µm. a.u.: arbitrary units.

We first examined if *bar-1* function was required cell-autonomously for D-type axon guidance, a necessary condition for the canonical pathway to act downstream of Wnt/Fz in this process. A functional BAR-1::GFP fusion protein [19] was expressed specifically in all GABAergic neurons, including the D-type motor neurons, using the *unc-47* promoter. This construct significantly rescued the D-type axon guidance defect (Fig. 5A), demonstrating a cell-autonomous role for β-catenin. This result is consistent with a previous report of endogenous *bar-1* expression in a subset of ventral nerve cord neurons [37].

β-catenin/BAR-1 is known to regulate cell fate decisions in a number of cells in *C. elegans*. To exclude the possibility that the observed defects in axon guidance were due to changes in cell fate in Wnt mutants, we carefully examined several features of D-type neurons. First, expression of the GABAergic specific marker *unc-47* was unchanged in all mutants analyzed as compared to wild-type animals, based on the expression of the *wyIs75* marker (Fig. 4A–H). In addition, the number, position and morphology of the DsRed-labeled cells in *wyIs75* animals were fully consistent with a D-type identity. In addition, a FMRF-like neuropeptide (*Pflp-13::GFP*, *ynIs37*) is normally expressed in the DD neurons and this expression is unaffected in *lin-44*, *bar-1* and *pop-1* mutants (Fig. 5C–E). Overall, we concluded that there is no evidence for a cell fate change of the D-type neurons in the Wnt-pathway mutants examined.

Finally, we tested whether LIN-17/Fz was acting solely through a β-catenin-dependent pathway to regulate axon guidance by examining *lin-17(n671); bar-1(ga80)* double mutant animals. If no other pathway was recruited downstream of Fz, we would expect the double mutant to show the same phenotype as the single *bar-1(ga80)* null allele. Instead, we observed that *lin-17(n671)*; *bar-1(ga80)* animals had intermediate phenotypes between *lin-17* and *bar-1* mutants (Fig. 5B). This result suggests that in addition to a β-catenin-dependent pathway, a different, non-canonical pathway is acting downstream of Wnt/Fz activation during axon pathfinding.

### Regulation of β-catenin by the ubiquitin-ligase F-box LIN-23 is required for correct axon guidance

**Figure 6:**
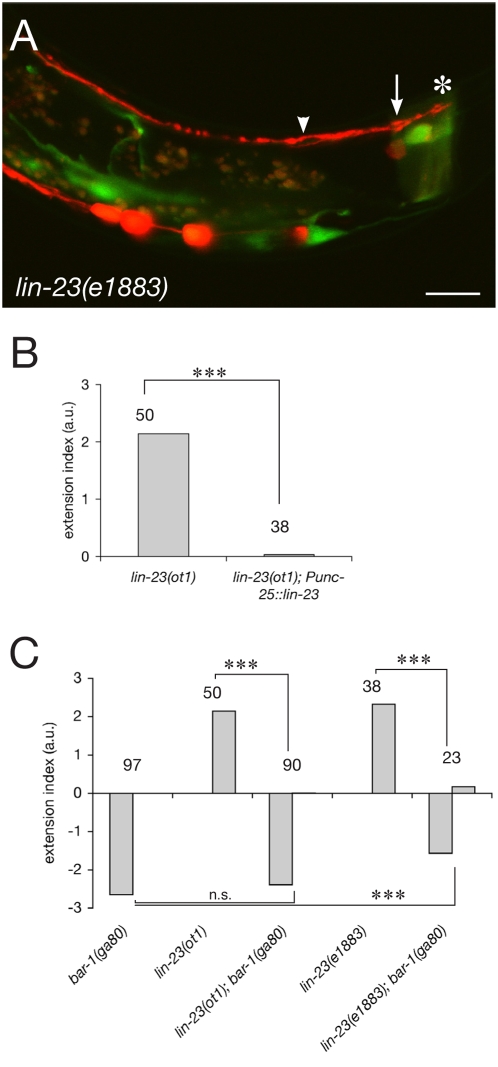
The ubiquitin ligase F-box LIN-23 regulates axon guidance through β-catenin. (A) In *lin-23* mutants, D-type axons overextend past the ADM (asterisk). (B) Expression of the *lin-23* cDNA specifically in D-type neurons is sufficient to completely rescue the overextension phenotype. ***p<0.001. (C) Extension index of *bar-1*; *lin-23* double mutants, compared to either of the single mutants. The double mutant shows a phenotype very similar to *bar-1* mutants, suggesting that *lin-23* regulates axon guidance through *bar-1*. n.s. p>0.1, ***p<0.001. Scale bar, 10 µm. a.u.: arbitrary units.

β-catenin can be targeted for degradation by the destruction complex through ubiquitin-mediated proteolysis [38]. In mammalian cells, the SCF^β-TrCP^ E3 ubiquitin ligase complex specifically recognizes β-catenin, among other substrates [39]. The *C. elegans* β-TrCP orthologue LIN-23 was also shown to interact with BAR-1 and regulate its stability [19]. We therefore asked whether loss of *lin-23*, which presumably leads to stabilization of β-catenin, would cause overextension of the D-type axons. Indeed, for two different loss-of-function alleles of *lin-23*, *ot1* and *e1883*, we found that D-type axons fail to terminate at their normal position (Fig. 6A,C). A similar overextension phenotype has been reported for the AVL neuron [40], a GABAergic motor neuron that has its cell body in the head and sends an axon along the ventral nerve cord up to the pre-anal ganglion, where the posterior DD-VDs reside. Intriguingly, in the same study, no endogenous expression of *lin-23* in commissural neurons was observed, raising the possibility of a non-cell autonomous effect of the *lin-23* mutation. We therefore tested if expressing the *lin-23* cDNA in D-type neurons (and in the RMEs, but not in AVL, DVB or RIS), using the *unc-25* minimal promoter [41], was sufficient to rescue their phenotype. In all lines examined, the overextension phenotype of the *lin-23(ot1)* mutant was robustly rescued (Fig. 6B). To confirm that the defects observed in *lin-23* mutants were due to a lack of BAR-1/β-catenin regulation, we constructed double *bar-1*; *lin-23* mutants. As expected, we found that the axon guidance phenotype of *lin-23(ot1)* mutants was completely suppressed by the *bar-1(ga80)* null allele (Fig. 6C). *bar-1*; *lin-23(e1883)* mutants show slightly milder underextension phenotypes than *bar-1* animals (Fig. 6C), which could be due to the excessive number of D-type neurons generated in *lin-23(e1883)* animals (*lin-23(ot1)* animals do not show this proliferation defect) [40].

## Discussion

In addition to its classical functions during early developmental patterning, the Wnt family of secreted glycoproteins has recently been shown to play a role during axon guidance (reviewed in [42]). However, the signal transduction pathway used to mediate this guidance effect is still unclear. The data presented here show that Wnts act as AP axon guidance cues in *C. elegans*, and that a β-catenin-dependent pathway is used downstream in D-type motor neurons to transduce this signal. Mutants for the Wnt ligands *lin-44* and/or *egl-20*, or for the Frizzled receptor *lin-17*, show D-type axon overextension defects along the AP axis. Ectopic expression of LIN-44/Wnt from dorsal body wall muscles induces D-type axons to stop prematurely after reaching the dorsal nerve cord, suggesting that Wnt is in fact an instructive guidance cue. Downstream of Wnt/Fz, we find that mutants for *mig-5*/Dsh, *gsk-3*, *pry-1*/Axin and *bar-1*/β-catenin also show D-type axon guidance defects, suggesting that Wnt-mediated axon guidance can be transduced through a β-catenin-dependent pathway.

### Wnts are conserved AP axon guidance cues

Studies in vertebrates, flies and worms have shown that Wnts are conserved axon guidance cues [43]. In *Drosophila*, the Ryk/Derailed receptor mediates repulsion of commissural axons by DWnt5 present in posterior commissures [3]. In both *C. elegans* and vertebrates, Wnts are conserved guidance cues along the AP axis, likely instructing the navigation of axons along the Wnt gradient to their appropriate AP position. Mammalian Ryk is used by corticospinal axons to grow caudally within the spinal cord, away from repulsive sources of Wnt [4]. In contrast, Fz receptors in vertebrates have so far been shown to mediate chemoattraction in response to Wnt [5], [6]. In *C. elegans*, however, all Wnts that guide axons have been shown to be repulsive through their interactions with Fz receptors [16], [17]. The worm Ryk orthologue *lin-18* might act redundantly with Fz-type receptors (this study and [16].

In this study, we show that Wnts are necessary for D-type axons to terminate at their appropriate AP position. As mentioned above, Wnts have been shown to act as repulsive cues for other neurons and could act in a similar manner in D-type neurons. However, our data is also consistent with Wnts acting as stop signals to induce D-type growth cone collapse. Closer examination of the growth cones behavior in the context of Wnt overexpression, and in the various Wnt-pathway mutants might shed light on the mechanism used by D-type axons.

### A β-catenin-dependent Wnt pathway regulates axon guidance downstream of Wnt/Fz

We show here that mutants for effectors downstream in the canonical β-catenin-dependent pathway display D-type axon guidance defects. These defects are either similar (*gsk-3* and *pry-1*/Axin) or opposite in nature (*mig-5*/Dsh, *bar-1*/β-catenin or *pop-1*/TCF) to those of Wnt mutants, suggesting that this pathway is at least partly responsible for mediating the axonal response to Wnts. This is supported by the fact that both the *lin-17*/Fz and *bar-1*/β-catenin mutant defects can be rescued cell-autonomously in D-type neurons. Furthermore, in mutants for the F-box/ubiquitin ligase *lin-23*, which has been shown to target BAR-1/β-catenin for degradation [19], D-type axons show phenotypes similar to those in *lin-44*/Wnt or *lin-17*/Fz mutant animals and opposite to those in *bar-1* mutant animals. This defect is both cell-autonomous and dependent on the presence of BAR-1.

**Figure 7:**
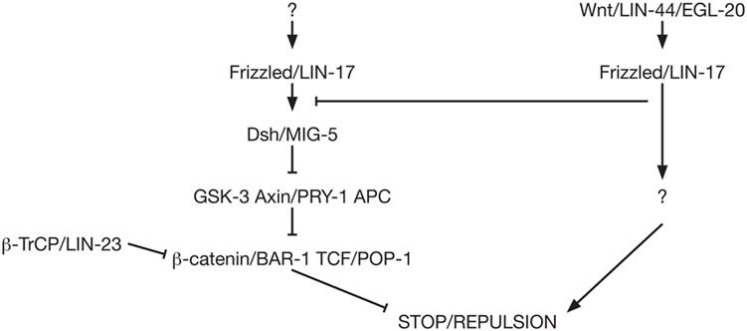
One possible model of the signaling pathways mediating axon guidance in response to Wnt. Because *lin-17* mutants have both underextension and overextension defects, we propose that LIN-17/Fz can activate both a canonical pathway that attracts axons (or prevents axon termination) (left) and a yet uncharacterized pathway that repels axons (or triggers axon termination) (right). The existence of this additional pathway and its interaction with the canonical pathway are supported by the analysis of *lin-17*; *bar-1* double mutants (see Figure 5). According to this model and consistent with our data, both the activation of the uncharacterized pathway and the inhibition of the canonical pathway by LIN-44/Wnt are required for the axons to stop in their correct position.

In addition to this β-catenin-dependent pathway, the analysis of *lin-17*; *bar-1* double mutants suggest that another, uncharacterized pathway is recruited downstream of LIN-44/Wnt and LIN-17/Fz in D-type neurons. One possible model for how these two pathways could interact to regulate axon guidance is presented in Figure 7. Because *lin-17*/Fz mutants show some underextension defects similar to *mig-5*/Dsh and *bar-1*/β-catenin mutants, in addition to their overextension phenotype, we propose that Fz/LIN-17 activates both a canonical pathway to stabilize β-catenin and prevent axon termination, and an uncharacterized pathway that triggers axon termination. This uncharacterized pathway could inhibit canonical signaling downstream of LIN-17/Fz, therefore reinforcing the repulsive effect of LIN-44/Wnt. This model would explain the surprising opposite phenotypes of *lin-17*/Fz and *mig-5*/Dsh mutants. It could also account for the lack of extension defects in DD's anterior to DD6, which are however able to respond to ectopic Wnt, in *bar-1* mutants. Indeed, these axons might be too far anterior to the source of Wnt in the tail, and thus be unable to activate the non-canonical pathway to promote termination. Interestingly, a recent study demonstrated that binding of Wnt-5a to the Ror2 receptor inhibits canonical Wnt-3a-induced signaling [44]. This inhibition, in contrast to what our model proposes however, happens downstream of β-catenin stabilization, at the level of target gene regulation.

How does β-catenin regulate axon guidance? In flies and vertebrates, β-catenin/Armadillo has a dual function in cell adhesion and transcriptional regulation [45]. In *C. elegans*, however, it is thought that these different functions are performed by distinct β-catenin paralogues [35]. We found that *bar-1*, which has been implicated in canonical Wnt signaling leading to transcriptional activation of target genes [25], [35], is required for correct axon guidance in D-type neurons. We also found that animals deficient for the β-catenin transcriptional co-activator POP-1/TCF had an underextension phenotype. It is therefore likely that activation of the Wnt pathway in D-type neurons leads to up- or downregulation of target genes that are themselves utilized during axonal outgrowth. One previously described example of transcriptional regulation coupled to axon guidance in vertebrates is the role of ZIC2 in retinal ganglion cells (RGCs) [46]. The transcription factor ZIC2 is expressed exclusively in neurons of the ventrotemporal retina, whose axons don't cross the midline but rather project ipsilaterally. ZIC2 is present when axons reach the midline and then downregulated as they navigate the optic tract and chiasm [46]. The axon guidance molecule EphB1 has also been shown to be required for proper ipsilateral projection of these RGCs [47]. It is thus likely that ZIC2 function is mediated at least in part by upregulation of EphB1in ipsilateral axons.

BAR-1 could also interact directly with the cytoskeleton to effect axonal remodeling. Indeed, in addition to its transcriptional function, the vertebrate β-catenin plays an essential role in cell adhesion by linking cadherins to the actin cytoskeleton, and interactions between these two functions have been suggested [48]. However, unlike HMP-2, one of the four β-catenin worm homologues, BAR-1 has not been implicated in cell adhesion thus far [35].

### Different Wnt pathways controlling axon guidance in distinct neurons

Our results point to the involvement of a β-catenin-dependent pathway downstream of Wnt/Fz in D-type axon guidance. However, we found that the phenotype of *lin-17* mutants was not completely suppressed by a *bar-1* mutation in D-type axons, suggesting that a second, β-catenin-independent pathway is involved in regulating axon guidance. In addition, we found that a different neuron, PDA, is misguided in *lin-44*/Wnt and *lin-17*/Fz mutants but not in mutants for other components of the canonical pathway, including *gsk-3*, *pry-1*/Axin and *bar-1*/β-catenin (G.S.M., data not shown). These data indicate that multiple Wnt pathways regulate axon guidance in *C. elegans*. Both the calcium pathway and the PCP pathway are excellent candidates and have been implicated in axon guidance in other model systems. Components of the calcium pathway, such as protein kinase C (PKC) or inositol-1,4,5 triphosphate (IP3), have been reported to mediate growth cone turning in cultured neurons [49]. Atypical PKC signaling has been implicated downstream of Wnt/Fz-mediated attraction of commissural axons [15]. In *Drosophila*, *DWnt4*, *DFz2* and *disheveled* are required for the attraction of ventral retinal axons, as is JNK, a kinase that acts in the PCP pathway [28]. JNK and Rac have also been implicated in Wnt-induced dendritic branching in cultured hippocampal neurons [13]. In addition, Dsh and GSK-3 can modulate microtubule stability by phosphorylating the microtubule-associated protein MAP1B [10]. Unfortunately, the degree of conservation of these pathways in nematodes is still unclear. Homologues of the PCP pathway components have recently been found to play a role in B cell division downstream of *lin-44*/Wnt and *lin-17*/Fz [50], and a potential role for these pathways in *C. elegans* axon guidance remains to be examined.

### Diversity of developmental processes regulated by Wnts

As discussed above, Wnts regulate a wide variety of biological processes, and in the *C. elegans* tail alone LIN-44/Wnt and LIN-17/Fz are required for asymmetric divisions of the T and B cells [22], [51], fate specification of the P12 cell [22], [52], HSN migration [16], polarity of the A/PLM mechanosensory neurons [17], [26], synaptic patterning in the DA9 motor neuron [18], and axon guidance of the D-type neurons as presented here. How can a single Wnt/Fz ligand/receptor pair trigger so many cellular responses? It has been proposed that the activation of different signal transduction cascades downstream of Wnt/Fz leads to different cellular outputs. For instance, B cell division utilizes a PCP-like pathway [50], whereas P12 cell fate specification is dependent on *bar-1*/β-catenin [53]. In disagreement with this simple model, however, some components of these pathways have been implicated in many diverse processes. This is the case for *bar-1*/β-catenin, which is required for P12 cell fate specification [53] and for D-type axon guidance (this work), as well as for *mig-5*/Dsh, which is used during QL neuroblast migration and P12 cell fate specification [54], B cell division [55], and by D-type axons (this work). It is thus likely that the particular developmental context of each cell, and not only the pathway that is recruited, influences the outcome of Wnt/Fz activation.

## Supporting Information

Figure S1 Schematic representation of the different classes of D-type axon termination phenotypes observed in mutants of the Wnt pathway. A score was given to each class, ranging from −3 to −1 for animals showing underextension (red, orange and yellow, respectively), 0 for wild-type animals (green), and from +1 to +3 for animals showing overextension (lighter to darker blue, respectively). The rationale for ordering the different categories was as follows: the yellow class was found in 20% of wild-type animals, thus prompting us to give this class the smallest score among the underextended categories; the red class was more severely affected than the orange class, resulting in their respective scores of −3 and −2. The dark blue class was obviously the most severe among overextending animals, and was thus given a score of +3. The light blue class was the major class of phenotypes found in addition to the wild-type ones in lin-23 or lin-17 rescued animals, and was thus given the smallest score among overextended categories, +1. As a result, the median blue class was given a score of +2.
(0.28 MB TIF)

Figure S2 Distribution of the phenotypes observed in various Wnt mutants, shown as percentages. The different classes of phenotype are presented in Supplementary Figure S1.
(0.51 MB TIF)

Figure S3 lin-17 act cell-autonomously in D-type neurons to regulate axon termination. (A,B) Distribution of the phenotypes observed in various Wnt receptor mutants shown as percentages (A) or as raw data (B). Because both underextension and overextension phenotypes can be observed for a given genotype, overextension and underextension indices were calculated separately in order to avoid averaging the opposing phenotypes. For instance, in lin-17 animals, the overextension and underextension indices were calculated as follows: iu = {32×(−3)+44×(−2)+0×(−1)} / 124 = −0.4, then normalized against wild type (−0.21, see Supplementary Fig. S2): −0.19 and io = {4×(+3)+0×(+2)+38×(+1)} / 124 = +1.48. (C) lin-17 mutant animals expressing a Punc-47::lin-17 construct show a robust rescue of the D-type axon overextension defect, as well as some underextension phenotypes. The different classes of phenotype are presented in Supplementary Figure S1.
(0.66 MB TIF)

Figure S4 Distribution of the phenotypes observed in various mutants of the canonical Wnt pathway, shown as percentages. The different classes of phenotype are presented in Supplementary Figure S1.
(0.44 MB TIF)

Figure S5 Mutants in the canonical Wnt pathway show D-type axon guidance defects at the L1 stage. (A–F) DD6 axon overextends in lin-44/Wnt (A), lin-17/Fz (B) and pry-1/Axin mutants (C), and underextends in mig-5/Dsh mutants (C), bar-1/β-catenin mutants (E) and pop-1/TCF mutants (F). The absence of extension defects in gsk-3 mutant L1 animals is consistent with the phenotypes observed in L4 animals, since class 1 and 3 phenotypes can be caused by overextension of VD13 axon only. In all images, the arrow indicates the wild-type termination point, and the asterisk indicates the abnormal termination point of DD6 axon. (G) Extension index for single and double mutants in components of the canonical Wnt pathway. Scale bar, 10 µm. a.u.: arbitrary units.
(8.25 MB TIF)
