# Supplementary figures and images for: A β-Catenin-Dependent Wnt Pathway Mediates Anteroposterior Axon Guidance in C. elegans Motor Neurons
Source: PLoS One. 2009 Mar 4;4(3):e4690. doi: 10.1371/journal.pone.0004690 (PMC2649571; doi:10.1371/journal.pone.0004690)

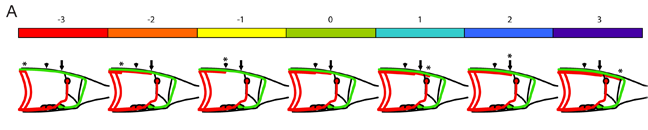

Supplement: Figure S1 — Schematic representation of the different classes of D-type axon termination phenotypes observed in mutants of the Wnt pathway. A score was given to each class, ranging from −3 to −1 for animals showing underextension (red, orange and yellow, respectively), 0 for wild-type animals (green), and from +1 to +3 for animals showing overextension (lighter to darker blue, respectively). The rationale for ordering the different categories was as follows: the yellow class was found in 20% of wild-type animals, thus prompting us to give this class the smallest score among the underextended categories; the red class was more severely affected than the orange class, resulting in their respective scores of −3 and −2. The dark blue class was obviously the most severe among overextending animals, and was thus given a score of +3. The light blue class was the major class of phenotypes found in addition to the wild-type ones in lin-23 or lin-17 rescued animals, and was thus given the smallest score among overextended categories, +1. As a result, the median blue class was given a score of +2. (0.28 MB TIF) [file pone.0004690.s001.tif]

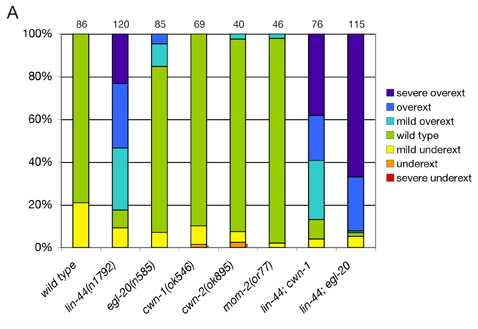

Supplement: Figure S2 — Distribution of the phenotypes observed in various Wnt mutants, shown as percentages. The different classes of phenotype are presented in Supplementary Figure S1. (0.51 MB TIF) [file pone.0004690.s002.tif]

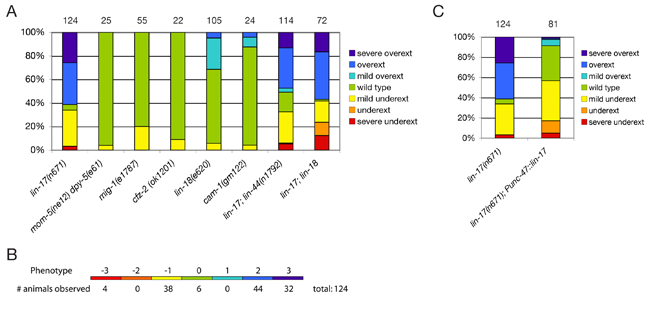

Supplement: Figure S3 — lin-17 act cell-autonomously in D-type neurons to regulate axon termination. (A,B) Distribution of the phenotypes observed in various Wnt receptor mutants shown as percentages (A) or as raw data (B). Because both underextension and overextension phenotypes can be observed for a given genotype, overextension and underextension indices were calculated separately in order to avoid averaging the opposing phenotypes. For instance, in lin-17 animals, the overextension and underextension indices were calculated as follows: iu = {32×(−3)+44×(−2)+0×(−1)} / 124 = −0.4, then normalized against wild type (−0.21, see Supplementary Fig. S2): −0.19 and io = {4×(+3)+0×(+2)+38×(+1)} / 124 = +1.48. (C) lin-17 mutant animals expressing a Punc-47::lin-17 construct show a robust rescue of the D-type axon overextension defect, as well as some underextension phenotypes. The different classes of phenotype are presented in Supplementary Figure S1. (0.66 MB TIF) [file pone.0004690.s003.tif]

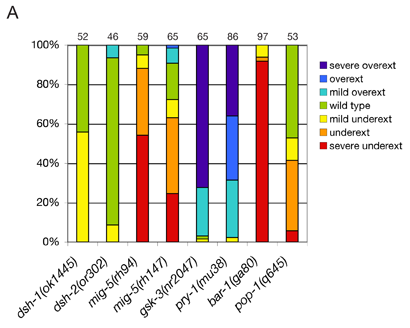

Supplement: Figure S4 — Distribution of the phenotypes observed in various mutants of the canonical Wnt pathway, shown as percentages. The different classes of phenotype are presented in Supplementary Figure S1. (0.44 MB TIF) [file pone.0004690.s004.tif]

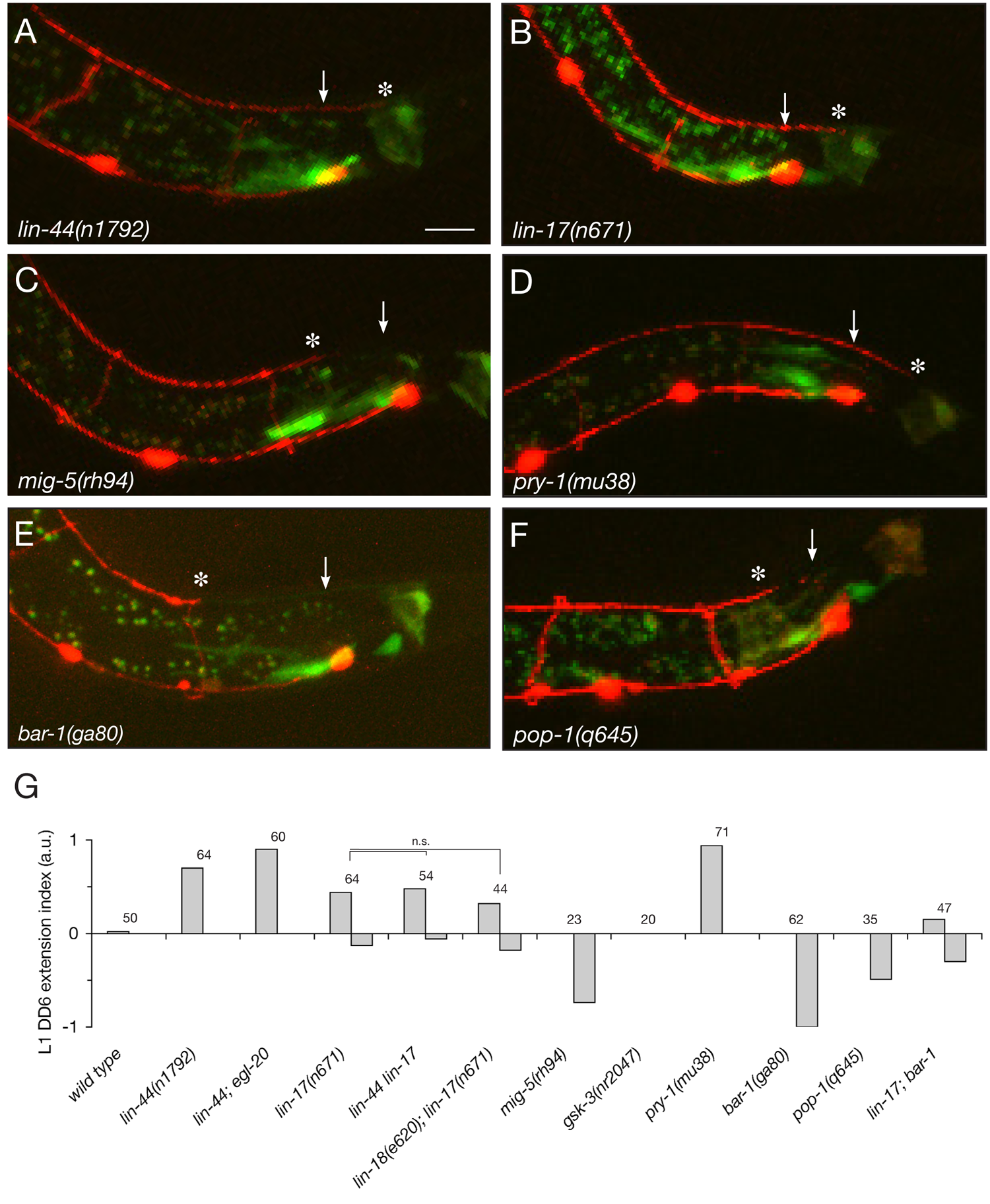

Supplement: Figure S5 — Mutants in the canonical Wnt pathway show D-type axon guidance defects at the L1 stage. (A–F) DD6 axon overextends in lin-44/Wnt (A), lin-17/Fz (B) and pry-1/Axin mutants (C), and underextends in mig-5/Dsh mutants (C), bar-1/β-catenin mutants (E) and pop-1/TCF mutants (F). The absence of extension defects in gsk-3 mutant L1 animals is consistent with the phenotypes observed in L4 animals, since class 1 and 3 phenotypes can be caused by overextension of VD13 axon only. In all images, the arrow indicates the wild-type termination point, and the asterisk indicates the abnormal termination point of DD6 axon. (G) Extension index for single and double mutants in components of the canonical Wnt pathway. Scale bar, 10 µm. a.u.: arbitrary units. (8.25 MB TIF) [file pone.0004690.s005.tif]
